# Ginkgolide C slows the progression of osteoarthritis by activating Nrf2/HO-1 and blocking the NF-κB pathway

**DOI:** 10.3389/fphar.2022.1027553

**Published:** 2022-10-28

**Authors:** Tianwen Ma, Lina Jia, Jinghua Zhao, Liangyu Lv, Yue Yu, Hongri Ruan, Xiaopeng Song, Hong Chen, Xin Li, Jiantao Zhang, Li Gao

**Affiliations:** ^1^ College of Veterinary Medicine, Northeast Agricultural University, Harbin, China; ^2^ College of Animal Science and Technology, College of Veterinary Medicine, Zhejiang Agriculture and Forestry University, Hangzhou, China; ^3^ Heilongjiang key Laboratory of Animals Disease Pathogenesis and Comparative Medicine, Harbin, China

**Keywords:** osteoarthritis, ginkgolide C, Nrf2/HO-1 pathway, NF-κB pathway, cartilage degeneration

## Abstract

Osteoarthritis (OA) is driven by chronic low-grade inflammation and subsequent cartilage degradation. OA is the most prevalent degenerative joint disease worldwide, and its treatment remains a challenge. The aim of this study was to explore the potential effects and mechanism underlying the anti-OA properties of ginkgolide C (GC). Protective effects of GC on hydrogen peroxide (H_2_O_2_)-treated rat chondrocytes were evaluated using ELISA, qPCR, western blot analysis, flow cytometry, ROS detection and immunofluorescence *in vitro*. Ameliorating effects of GC on cartilage degeneration in rats were evaluated through behavioral assays, microcomputed tomography, histopathological analysis, western blot analysis and ELISA *in vivo*. *In vitro*, GC treatment inhibited the release of pro-apoptotic factors induced by H_2_O_2_ and promoted the release of the anti-apoptotic proteins. In addition, GC decreased the expression of matrix metalloproteinase (MMP3 and MMP13), thrombospondin motifs 4 (ADAMTS4), and inflammatory mediators inducible nitric oxide synthase (iNOS), cyclooxygenase (COX-2), and SOX9 thereby inhibiting extracellular matrix (ECM) degradation. Mechanistically, GC exerts its anti-apoptotic and anti-inflammatory effects by upregulating the oxidative stress signaling Nrf2/HO-1 pathway and preventing p65 from binding to DNA. Similarly, In a rat model with post-traumatic OA (PTOA) induced by anterior cruciate ligament transection (ACLT), GC inhibited joint pain, cartilage destruction, and abnormal bone remodeling of subchondral bone. GC inhibited H_2_O_2_-induced chondrocyte apoptosis through Nrf2/HO-1 and NF-κB axis, exerted anti-inflammatory effects, and inhibited cartilage degeneration in rat OA. Our findings advanced the concept that GC may contribute to cartilage metabolism through anti-inflammatory and anti-apoptotic effects, and the identified GC is a potential therapeutic agent for the treatment of OA.

## 1 Introduction

Osteoarthritis (OA) is a chronic aggressive inflammatory disease characterized by progressive articular cartilage erosion, the imbalance in extracellular matrix (ECM) synthesis and degradation, subchondral sclerosis, osteophyte formation, and synovial hyperplasia (Ashford and Williard, 2014). Unfortunately, the therapeutic options are limited by high toxicity and intolerable side effects (Magni et al., 2021). Therefore, despite many advances in novel therapies for OA, there is a need to develop new therapeutic agents with low toxicity and minimal side effects.

Results from studies using herbal and plant extracts to manage OA are encouraging and may provide greater flexibility in clinical settings (Gao et al., 2021; Mu et al., 2021). *Ginkgo biloba* L. extract is widely used worldwide as the first choice botanical medicine for the treatment of heart and brain diseases (Abad et al., 2010). Ginkgolide C (GC), isolated from *Ginkgo biloba* L. extract, is a flavonoid with various biological functions (Fang et al., 2020). GC has a wide range of pharmacological effects, such as scavenging free radicals (Wang et al., 2021), regulating neurotransmitter and hormone levels (Feng et al., 2019), improving blood rheology (Liang et al., 2022), and anti-inflammatory effects (Zhang et al., 2021). Studies have shown that *Ginkgo biloba* L. prevents chondrocyte degeneration by inhibiting JNK activation and causing ubiquitin-dependent c-Jun degradation (Ho et al., 2013). *Ginkgo biloba* L. has anti-inflammatory effects on human articular chondrocytes and OA rats (Chen et al., 2013). The main components of *Ginkgo biloba* L. extract, bilobalide and ginkgolide B, have been reported to reverse OA (Hu et al., 2018; Mao et al., 2019; Ma et al., 2022a). However, the biological function of GC on cartilage has not been reported.

Oxidative stress is not only associated with chronic inflammation but also plays a major role in the pathophysiology of OA (Tudorachi et al., 2021). Nuclear factor-erythroid 2-related factor-2 (Nrf2) is a nuclear transcription factor involved in the expression of various proteins and is the regulatory center of the body’s oxidative stress response (Bolduc et al., 2019). The production of several downstream antioxidants, anti-inflammatory proteins, and detoxification enzymes can be triggered when Nrf2 binds to the antioxidant response element (ARE), helping to keep the body’s redox balance in check. Heme oxygenase-1 (HO-1) activity controls crucial biological processes such as cell proliferation, inflammation, fibrosis, and angiogenesis. HO-1 gene expression is connected to Nrf2 (Sanada et al., 2021). On the other hand, the nuclear factor kappa B (NF-κB) signaling pathway is a downstream effector of inflammatory cytokines that may stimulate inflammation and catabolism in chondrocytes (Oeckinghaus et al., 2011). Emerging research shows that modulating the Nrf2/HO-1 and NF-κB signaling axis could counteract oxidative stress, inflammation, and apoptosis in multiple diseases (Zhang Y. et al., 2022; Zhang Y. Y. et al., 2022; Luo et al., 2022).

There are recent discoveries in the chemistry and mechanism of action of compounds in *Ginkgo biloba* L. extract (Ma et al., 2022a; Zhang R. et al., 2022; Samec et al., 2022). However, not many reports highlight the benefit of GC on degenerative cartilage diseases. This study investigated the protective effect of GC derived from *Ginkgo biloba* L. extract on OA in rats and explained its possible mechanism based on the Nrf2/HO-1 and NF-κB axis. Furthermore, we provided new insights into clinical application of *Ginkgo biloba* L. extract.

## 2 Materials and methods

### 2.1 Reagents and antibodies

Ginkgolide C (GC, Purity ≥98%, PubChem CID: 161120) was purchased from Chengdu Must Bio-Technology Co., Ltd. (Chengdu, China). Zinc (II) Protoporphyrin IX (ZnPP), protease Inhibitor and ML385 were purchased from MCE (MedChemExpress, United States). Toluidine blue and 4% paraformaldehyde were purchased from Solarbio (Beijing, China). Type II Collagenase and 0.25% Trypsin-EDTA were purchased from Gibco (United States). Penicillin/streptomycin, CCK-8, DAPI, and fluorescent probe DCFH-DA were purchased from Beyotime (Shanghai, China). ProteinTech (Wuhan, China) provided the main antibodies for MMP3, IκBα, GAPDH, HO-1, and Nrf2. p65, Bcl-2, SOX9, and cleaved-caspase3 (C-caspase3) antibodies were bought from Cell Signaling Technology (Danvers, United States). Antibodies for Bax, iNOS, MMP13, Nrf2, p-IκBα, and Lamin B were bought from Affinity (Jiangsu, China). Antibodies for COX-2 and ADAMTS4 were acquired from ABclonal (Wuhan, China). OriGene (Wuxi, China) provided the β-actin antibody. Alexa 488 Goat Anti-Rabbit IgG (H + L) was purchased from APExBIO (Houston, United States).

### 2.2 Isolation and culture of rat primary chondrocytes

Primary chondrocytes from rats were treated using a previously described protocol (Bai et al., 2020). Briefly, Sprague Dawley rats that were 14–21 days old had their proximal tibia and distal femur articular cartilage harvested. For 30 min articular cartilage was digested with 0.25% Trypsin-EDTA. The fresh cartilage samples were then divided into pieces of about 1 mm^3^ and digested for 4–6 h with 0.2% type II collagenase. Cells were plated in 25 cm gas-permeable culture flasks containing 10% fetal bovine serum (FBS, ExCell Bio, China) in DMEM/F12 and 1% penicillin/streptomycin, then cultured at 37°C in an incubator containing 5% CO_2_. Next, Toluidine blue and Allison blue staining for proteoglycans were used to identify chondrocytes. The second-generation rat chondrocytes were taken for the following experiments to avoid phenotype loss.

### 2.3 Cell viability

The cytotoxicity of GC and H_2_O_2_ on chondrocytes was measured using a cell counting kit (CCK-8) assay according to the manufacturer’s protocol. Primary rat chondrocytes were cultured in 96-well plates (5000/well) for 24 h and then incubated with varying concentrations of GC (0, 7.5, 15, 30, 60, and 120 μM) for 24 h, respectively. To test the optimal dose of H_2_O_2_, chondrocytes were cultured with H_2_O_2_ (0, 0.1, 0.2, 0.3, 0.4, and 0.5 mM) alone in DMEM/F12 medium for 24 h. Additionally, DMEM/F12 medium with or without H_2_O_2_ was added to the co-apply with chondrocytes and incubated in different concentrations of GC for 24 h to determine the effective concentrations of hydrogen peroxide and GC to stimulate chondrocytes. Then, 100 μL of DMEM/F12 supplemented with 10 μL of CCK-8 solution was added to each well and incubated at 37°C for 30 min. The absorbance of each well was measured at 450 nm using a multi-plate reader (BioTEK, United States).

### 2.4 Measurement of reactive oxygen species

Chondrocytes were seeded in 6 cm dishes and treated with different concentrations of GC and/or H_2_O_2_ for 24 h. The intracellular ROS were measured after cells were treated with fluorescent probe dichlorodihydrofluorescein diacetate (DCFH-DA) at 37°C for 30 min. The fluorescence intensity of DCF was measured using a fluorescence microscope (Leica, Germany).

### 2.5 Immunofluorescence

Cells were seeded in confocal dishes (35 mm), then cultured for 24 h in cultured chondrocytes with or without H_2_O_2_ and GC. Chondrocytes were washed three times with PBS, fixed with 4% paraformaldehyde for 20 min, and then infiltrated using 0.5% Triton X-100 for 20 min at room temperature. Next, cells were blocked with 3% BSA for 30 min. Then p65 antibody was added to confocal dishes and incubated overnight at 4°C. Next, cells were incubated with the Alexa 488 Goat Anti-Rabbit IgG (H + L) antibody for 1 h at room temperature and stained with DAPI for 10 min at room temperature. Finally, the cells were added to the anti-fluorescence quenching mounting solution and observed under a fluorescence microscope (Leica, Germany).

### 2.6 Determination of cell apoptosis

Annexin V/PI apoptosis assay (Abbkine, China) was conducted after different treatments. Briefly, chondrocytes were washed three times with PBS and harvested after trypsinization. Then cells were resuspended in 100 µL of 1x Annexin V Binding Buffer. Next, 4–5 µL Annexin V-AbFlourTM 488 and 1–2 µL PI per 100 µL of cell suspension were added and mixed gently. The reaction was carried out in the dark for 15 min. After the incubation, 400 µL of 1 × Annexin V Binding Buffer was added. Chondrocyte apoptosis was then evaluated within 30 min using the flow cytometry (BD, United States), and the data were analyzed using the FlowJo software. The cell apoptosis was detected by the TUNEL method (Abbkine, China). The chondrocytes in each group were incubated with TUNEL solution at 4°C for 1 h and incubated with DAPI for nuclear staining. Fluorescence microscopy was used to find and record fluorescence pictures in all groups.

### 2.7 Quantitative real-time polymerase chain reaction analysis

The total RNA of chondrocytes of each group in the cell culture incubator was extracted using a RNAsimple total RNA kit (Tiangen, China) according to the manufacturer’s instructions. Reverse transcription was performed using the ReverTra Ace qPCR RT Master Mix with gDNA Remover (Vazyme, China). Complementary DNA (cDNA) was amplified using the ChamQ Universal SYBR qPCR Master Mix (Vazyme, China). Using the relative quantification technique (2−^ΔΔCt^ method), the expression levels of the target genes were assessed in comparison to -actin levels. Table 1 contains the forward and reverse primer sequences for each gene.

**TABLE 1:** The primer sequences used in qPCR assay.

Gene	Forward primer (5′–3′)	Reverse primer (5′–3′)
iNOS	AAG​AGA​CGC​ACA​GGC​AGA​GG	AGC​AGG​CAC​ACG​CAA​TGA​TG
SOX-9	CTC​CCA​AAA​CAG​ACG​TGC​AA	CGA​AGG​TCT​CGA​TGT​TGG​AGA​T
COX-2	AGA​AGC​GAG​GAC​CTG​GGT​TCA​C	ACA​CCT​CTC​CAC​CGA​TGA​CCT​G
MMP3	TTT​GGC​CGT​CTC​TTC​CAT​CC	GCA​TCG​ATC​TTC​TGG​ACG​GT
MMP13	TTC​TGG​TCT​TCT​GGC​ACA​CG	TGG​AGC​TGC​TTG​TCC​AGG​T
ADAMTS4	AGG​AGG​CGC​CCT​TAA​CTC​TG	CTA​CTC​AGC​GAA​GCG​AAG​CG
Bax	TGA​AGA​CAG​GGG​CCT​TTT​TG	AAT​TCG​CCG​GAG​ACA​CTC​G
Caspase-3	GCT​TGG​AAC​GGT​ACG​CTA​AGA	CCC​AGA​GTC​CAC​TGA​CTT​GC
Bcl2	GCT​ACC​GTC​GTG​ACT​TCG​C	CCC​CAC​CGA​ACT​CAA​AGA​AGG
β-actin	GTG​ACG​TTG​ACA​TCC​GTA​AAG​A	GCC​GGA​CTC​ATC​GTA​CTC​C

This is a provisional file, not the final typeset article.

### 2.8 Establishment of a rat model of post-traumatic osteoarthritis

Fifty male Sprague-Dawley rats aged 11–12 weeks were purchased from Changchun Yisi Experimental Animal Technology Co., Ltd. (Changchun, China). A PTOA rat model was established using the standard anterior cruciate ligament transection (ACLT) method according to a previous protocol (Ma et al., 2018). Briefly, ACLT surgery was performed under anesthesia with isoflurane. Rats were randomly divided into five groups (n = 10 in each group): control group (sham surgery), OA group (ACLT surgery-induced OA), celecoxib group (2.86 mg/kg body weight celecoxib (Bai et al., 2020) as positive dosing control), GC-L group (rats treated with 5 mg/kg body weight GC) and GC-H group (rats treated with 10 mg/kg body weight GC). The rats in the GC-L group and the GC-H group were given 5 and 10 mg/kg of GC by gavage every day, respectively. Requirements considered to be relevant in guidelines for best practice in natural products pharmacological research have been taken into account (Heinrich et al., 2020). GC was prepared in sterile saline solution containing 2% Tween-80. Two weeks after ACLT surgery, rats in the control group and OA group were given 2 ml of sterile saline solution containing 2% Tween-80, and rats in the GC-L group and the GC-H group were given 5 and 10 mg/kg of GC by gavage every day, respectively. After continuous administration for 6 weeks, the rats in each group were euthanized by a lethal dose of ether *via* intraperitoneal injection. Next, the knee joint and blood samples were taken. Then blood was centrifuged at 1000 g for 20 min at room temperature. All rats were performed in accordance with the guide for the Care and Use of Laboratory Animals of National Institutes of Health (Kastenmayer et al., 2012). The animal surgery procedure and treatments used in this study were approved by the Laboratory Animal Welfare and Ethics Committee of Northeast Agricultural University (#NEAU-2021-03-115). Every effort was made to reduce animal suffering and reduce the number of animals used.

### 2.9 Knee width measurement

The knee joint width of each group of rats was measured using an electronic digital caliper to assess the degree of joint swelling (Sudirman et al., 2020). Right knee joint width in rats was measured before ACLT surgery (baseline) and remeasured weekly until the end of the experiment. The same researcher performed the knee width measurements to reduce errors.

### 2.10 Mechanical sensitivity

Mechanical sensitivity in rats was measured before ACLT surgery (baseline) and remeasured weekly until the end of the experiment. Mechanical sensitivity was assessed as previously described by measuring the withdrawal thresholds of both hind paws in response to the application of von Frey filaments using the up-down method (Katri et al., 2019).

### 2.11 Cold hypersensitivity

The rat was placed on a wire mesh floor, acetone was applied to the center of the animal’s hind paw, and a stopwatch was started. Rats that showed no response (stamp, flick, or paw withdrawal) were given a score of 0. Scores were recorded within 20 s of the application of acetone according to the previous scoring scale (1–4 scores) (Katri et al., 2019). Three acetone tests per paw were performed with at least 10 min between the tests, and then the scores were summed. Cold hypersensitivity was assessed at the baseline and within 8 weeks postoperatively.

### 2.12 Western blotting

Total protein was extracted from chondrocytes using RIPA containing protease inhibitor. The chondrocyte nuclear protein and plasma protein extraction were carried out using Nuclear protein and Cytoplasmic Protein Extraction Kit (Beyotime, China). In addition, at the end of 8 weeks of ACLT surgery, the dissected cartilages were removed from the right knee joint of the rat and homogenized in 1 ml PBS (pH 7.4) containing protease inhibitors by shaking with 1.5-mm glass beads in a Mini-BeadBeater (BioSpec, Bartlesville, OK) at 4800 oscillations/min for 3 min. The tissue homogenates were immediately centrifuged twice at ×12,000 g for15 min at 4°C to isolate supernatant for subsequent western blot abalysis. The same quantity of protein was separated using 8%–12% SDS–PAGE gels after the determination of the protein concentration using the BCA kit (Beyotime, China) and balancing. SDS-PAGE was used to separate the proteins, and then the protein bands were transferred to PVDF membranes. PVDF membranes were incubated with the appropriate antibodies overnight at 4°C after blocking with 5% skimmed milk or 5% BSA powder soaked in TBST for 2 h. The membranes were subsequently incubated with secondary antibody for 1 h at room temperature. Western blots were visualized using an ECL chemiluminescence reagent (Vazyme, China). Protein bands were obtained using the Tannon automated gel image analysis system (Shanghai, China). ImageJ software was used to analyze the relative expression levels of proteins.

### 2.13 SiRNA and cell transfection

First, siRNA negative control (si-Crtl) and its target siRNA Nrf2 (si-Nrf2) by Ribobio (Guangzhou, China) were constructed and synthesized. Then, chondrocytes were seeded in a 6-well plate for 24 h. Transfection with si-Nrf2 or si-NC were carried out using Lipofectamine 2000 reagent according to the protocol.

### 2.14 Microcomputed tomography (μCT)

Tibial samples from each group were scanned using Bruker MicroCT Skyscan 1276 system (Kontich, Belgium). Scan settings are as follows: voxel size 10.133345μm, medium resolution, 85 kV, 200 mA, 1 mm Al filter and integration time 525 ms. Density measurements were calibrated to the manufacturer’s calcium hydroxyapatite (CaHA) phantom. Analysis was performed using the manufacturer’s evaluation software. Reconstruction was accomplished by NRecon (version 1.7.4.2). 3D images were obtained from contoured 2D images by methods based on distance transformation of the grayscale original images (CTvox; version 3.3.0). 3D and 2D analysis were performed using software CT Analyser (version 1.18.8.0). Analyses of the bone micro architecture were carried out in a region of interest (ROI). Quantitative analysis of BV/TV, mean Tb.N, mean Tb.Th and mean Tb. Sp were measured for each sample.

### 2.15 Hematoxylin-eosin and saffron O fast green staining

Each rat’s tibia and femur samples were first fixed in 10% neutral buffered formalin, followed by paraffin embedding. The blocks were cut into sagittal sections with a thickness of 5 μm. Then the cartilage matrix was stained with SO-FG while HE was used to evaluate the pathological changes to the cartilage. The Osteoarthritis Research Society International (OARSI) scoring system (Pritzker et al., 2006) was used to assess the degree of pathological damage to the knee cartilage.

### 2.16 ELISA detection of rat osteoarthritis biomarkers

The C-terminal peptide levels of C-telopeptides of type II collagen (CTX-II) (Mlbio, China) and Cartilage oligomeric matrix protein (COMP) (Mlbio, China) in rat serum were determined strictly according to the instructions of commercial ELISA kits. Three replicate wells were used for each set of samples, and average OD values were obtained using a multi-plate reader.

### 2.17 Statistical analysis

Statistical analyses were performed using SPSS software (version 19.0 for Windows, SPSS, Chicago, IL, United States). The results are summarized as mean ± standard deviation (SD). For two-group comparisons, data were analyzed by a two-tailed Student’s *t*-test. For multiple group comparisons, statistical analysis was performed by ANOVA followed by least significant difference (LSD) test. *p* value <0.05 was considered statistically significant.

## 3 Results

### 3.1 Effect of ginkgolide C on chondrocyte viability

**FIGURE 1:**
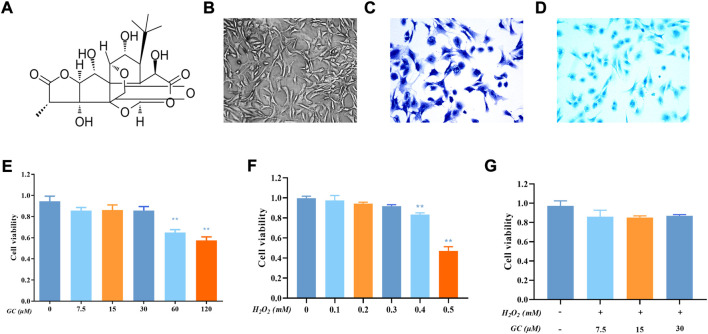
Effect of Ginkgolide C (GC) on chondrocyte viability with or without H_2_O_2_. **(A)** Chemical structure of the GC. **(B)** Representative image of second-generation chondrocytes rat chondrocyte morphology. **(C)** Representative image of Toluidine blue staining. **(D)** Representative image of Allison blue staining. **(E)** Cell Counting Kit-8 (CCK-8) detected the cell viability of chondrocytes with different concentrations of GC for 24 h **(F)** CCK-8 results for chondrocytes treated with different concentrations of hydrogen peroxide (H_2_O_2_) for 24 h **(G)** CCK-8 results for GC-treated chondrocytes stimulated with H_2_O_2_. All data are presented as mean ± SD (n = 3). **p < 0.01 relative to the control group.

The structure of GC is shown in Figure 1A. The second-generation chondrocytes showed irregular polygonal shape, complete nuclei and proliferated rapidly (Figure 1B). Rat second-generation chondrocytes were used in all subsequent experiments. The proteoglycans in the extracellular matrix of cartilage were stained bluish-violet and blue with Toluidine blue (Figure 1C) and Allison blue (Figure 1D). The CCK-8 kit was used to detect the toxicity of GC to chondrocytes. First, we utilized 7.5, 15, and 30 μM GC to intervene chondrocytes in future tests since the vitality of chondrocytes was drastically decreased when the GC concentration reached 60 μM (Figure 1E). On the other hand, chondrocyte viability is drastically reduced when H_2_O_2_ concentration exceeds 0.4 mM, thus we employ this concentration to cause chondrocyte injury (Figure 1F). Chondrocyte viability was not significantly changed upon simultaneous addition of GC and 0.4 mM H_2_O_2_, as seen in Figure 1G. These findings demonstrate that GC protects injured chondrocytes.

### 3.2 Ginkgolide C inhibits matrix-degrading protein production and chondrocyte apoptosis in H_2_O_2_-induced chondrocytes

**FIGURE 2:**
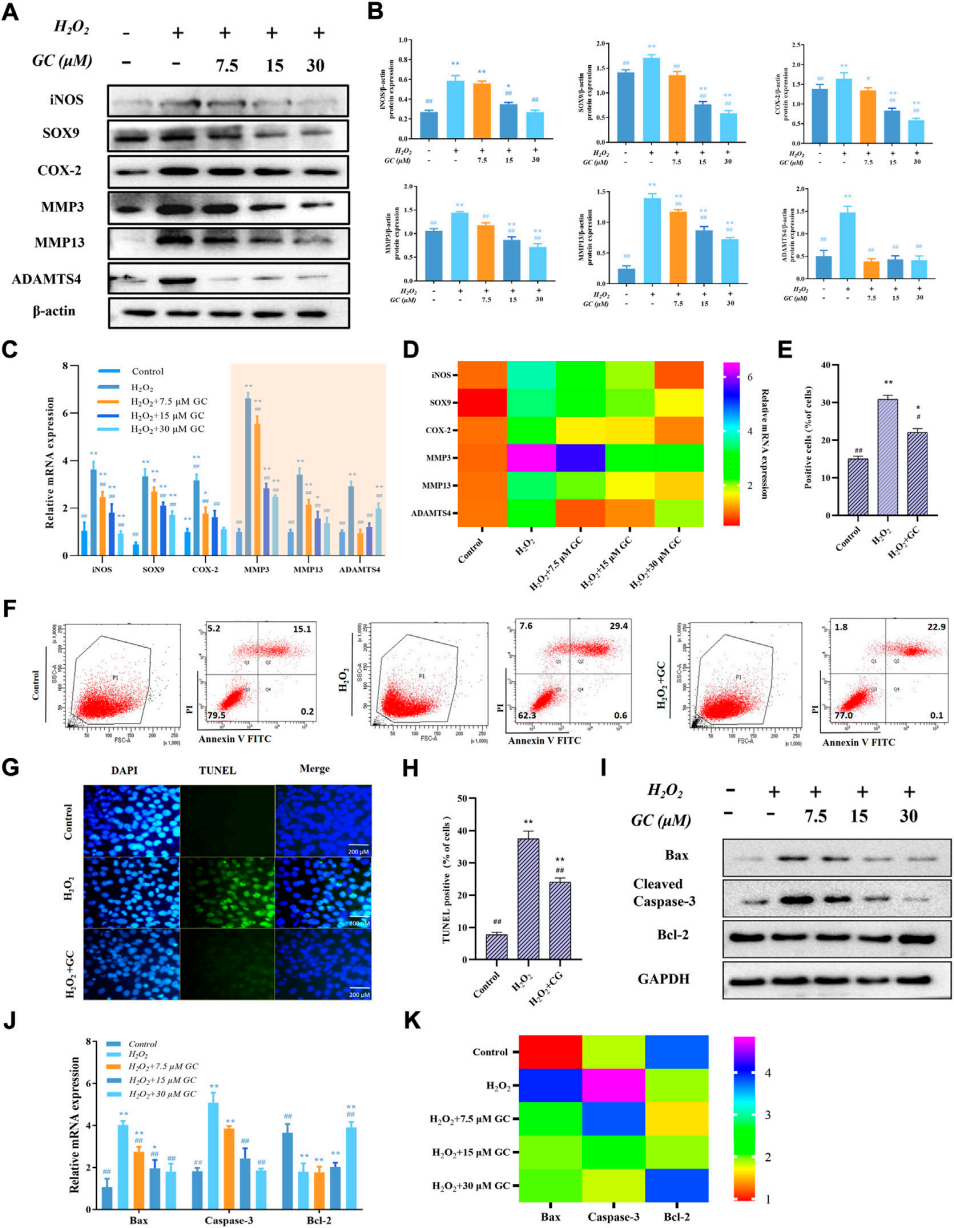
GC inhibits H_2_O_2_-induced chondrocyte apoptosis and cartilage degradation. **(A)** By using a western blot, it was possible to identify the protein expression of iNOS, COX-2, SOX-9, MMP3, MMP13, and ADAMTS4 in chondrocytes that had been exposed to GC and H_2_O_2_ for 24 h. **(B)** Using ImageJ, the related protein expression was examined. **(C and D)** The expression levels of certain cartilage degeneration-related genes were evaluated using qPCR. **(E and F)** Annexin V/PI detected GC on H_2_O_2_-induced chondrocyte apoptosis. When calculating the apoptosis rate, the sum of the apoptosis rates in the Q2 and Q4 quadrants was used, that is, the late apoptotic group + the early apoptotic group (that is, all AnnexinV positive groups, of which the Q2 quadrant was the PI staining positive group). **(G)** Examples of terminal deoxynucleotidyl transferase-positive, deoxyuridine triphosphate nick end-labeled nuclei (green color). 200 µm scale bar. **(H)** Percentages of TUNEL-positive cells relative to total cells. **(I)** Western blot analysis was used to identify the protein expression of Cleved-caspase 3, Bax, and Bcl-2 in chondrocytes co-applied with GC and H_2_O_2_ for 24 h **(J and K)** The expression levels of certain apoptosis-related genes were evaluated using qPCR. All data are presented as mean ± SD (n = 3). *p < 0.05 and **p < 0.01 *vs.* control group; ^#^ *p* < 0.05 and ^##^ *p* < 0.01 *vs.* H_2_O_2_ group.

H_2_O_2_ is one of the most frequent triggers for chondrocyte damage because it exposes chondrocytes to an environment of oxidative stress, which leads to cartilage degradation and chondrocyte apoptosis (Luo et al., 2022; Zhou et al., 2022). Western blot and qPCR were used to examine the impact of GC on the inflammatory response and extracellular matrix breakdown of rat chondrocytes stimulated by oxidative stress caused by H_2_O_2_. Induction of chondrocytes with H_2_O_2_ led to aberrant elevations in the levels of the proteins iNOS, COX-2, SOX-9, MMP3, and ADAMTS4, as seen in Figures 2A,B. However, GC could counteract these effects. Similarly, as shown in Figures 2C,D, in H_2_O_2_-induced chondrocytes, GC intervention inhibited the expression of iNOS, COX-2, SOX-9, MMP3, MMP13, and ADAMTS4 mRNA. We used flow cytometry, TUNEL, western blotting, and qPCR to examine the impact of GC on the apoptosis of rat chondrocytes stimulated by oxidative stress caused by H_2_O_2_. As shown in Figures 2E,F, according to the flow cytometry quantification, the fraction of apoptotic cells was decreased in the 30 μM GC group compared to H_2_O_2_ treatment alone. The rate of TUNEL-positive cells was also raised during H_2_O_2_ stimulation and reversed by 30 μM GC, as shown in Figures 2G,H, demonstrating that GC shielded rat chondrocytes against oxidative stress-induced apoptosis. Additionally, western blot analysis revealed that GC treatment decreased the expression of Cleaved-caspase-3 and Bax and reversed the decrease in Bcl-2 brought on by H_2_O_2_ (Figure 2I and Supplementary Figure S1). It was further confirmed using qPCR that GC treatment decreased Caspase-3 and Bax mRNA expression and increased Bcl-2 mRNA expression (Figures 2J,K). These data indicate that GC exerts anti-apoptotic and anti-chondral degradation effects in rat chondrocytes.

### 3.3 Ginkgolide C inhibited ROS generation by activating the Nrf2/HO-1 signaling pathway in chondrocytes

**FIGURE 3:**
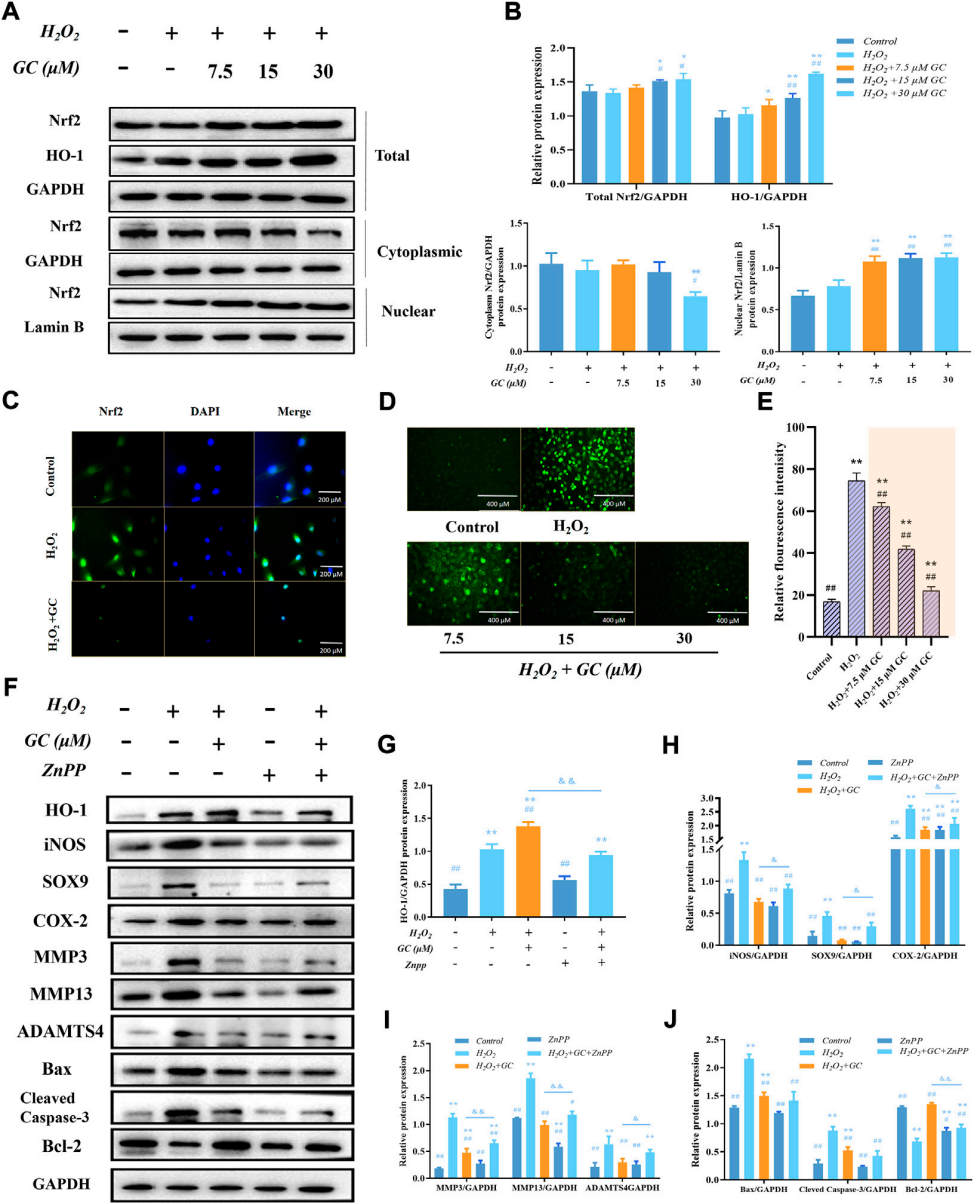
The expression of HO-1 in chondrocytes that have been exposed to H_2_O_2_ is linked to the anti-apoptotic and anti-chondral degrading effects of GC. **(A and B)** Western blot images and quantification information for the protein expression of the Nrf2/HO-1 signaling pathway in each group following the aforementioned treatment. **(C)** Immunofluorescence detection of nuclear translocation of Nrf2 protein with DAPI staining for nuclei. 200 µm scale bar. **(D and E)** Dichlorodihydrofluorescein diacetate (DCFH-DA) fluorescent staining showed that GC inhibited the H_2_O_2_ induced ROS production. 400 µm scale bar. **(F–J)** Western blot imaging and quantification data for each group treated with H_2_O_2_, GC, and 10 μM Zinc (II) Protoporphyrin IX (ZnPP) for HO-1, iNOS, SOX9, COX-2, MMP3, MMP13, ADAMTS4, Bcl-2, Bax, and Cleved-caspase 3. All data are presented as mean ± SD (n = 3). *p < 0.05 and **p < 0.01 *vs.* control group; ^#^ *p* < 0.05 and ^##^ *p* < 0.01 *vs.* H_2_O_2_ group; ^＆^ *p* < 0.05 and ^＆＆^ *p* < 0.01 vs. H_2_O_2_ + GC group.

We speculate that the molecular mechanism by which GC alleviates H_2_O_2_-activated cartilage oxidative damage may be related to the Nrf2/HO-1 pathway. As shown in Figures 3A,B, Western blot results showed that the expressions of total proteins Nrf2 and HO-1 increased with increasing GC dose. After H_2_O_2_-induced chondrocytes were treated with GC, the expression of Nrf2 in the cytoplasm was decreased. In contrast, the expression of Nrf2 in the nucleus was increased. The nuclear translocation of Nrf2 was determined by immunofluorescence assay to explain the effect of 30 μM GC on the H_2_O_2_-induced Nrf2/HO-1 signaling pathway in chondrocytes (Figure 3C). We observed a higher degree of Nrf2 nuclear translocation when GC was cultured with H_2_O_2_. We used fluorescent probe DCFH-DA fluorescent staining to determine the influence of GC on ROS overproduction when co-applied with H_2_O_2_ (Figures 3D,E). The green fluorescence intensity was significantly increased following H_2_O_2_ treatment alone, and the expression of ROS in chondrocytes was dose-dependently decreased after GC intervention. The above results suggested that GC inhibits ROS generation in chondrocytes by activating the Nrf2/HO-1 signaling pathway.

### 3.4 The expression of HO-1 is linked to the anti-apoptotic and anti-chondral degradation effects of ginkgolide C

In order to determine if GC induces the production of HO-1 in order to exert its anti-apoptotic and anti-chondral degradation impact, firstly, we have demonstrated in previous experiments that the expression of HO-1 increases with increasing GC dose. After that, chondrocytes were treated with the HO-1 inhibitor 10 µM ZnPP, and the findings of a western blot demonstrated that ZnPP was able to reverse GC’s protective impact on chondrocytes as well as its capacity to prevent apoptosis and prevent chondral deterioration (Figures 3F–J). All of these findings imply that the enhanced production of HO-1 contributes to the anti-apoptotic and anti-chondral degradation of GC in rat chondrocytes treated with H_2_O_2_.

### 3.5 Nrf2 nuclear translocation boosts HO-1 expression

**FIGURE 4:**
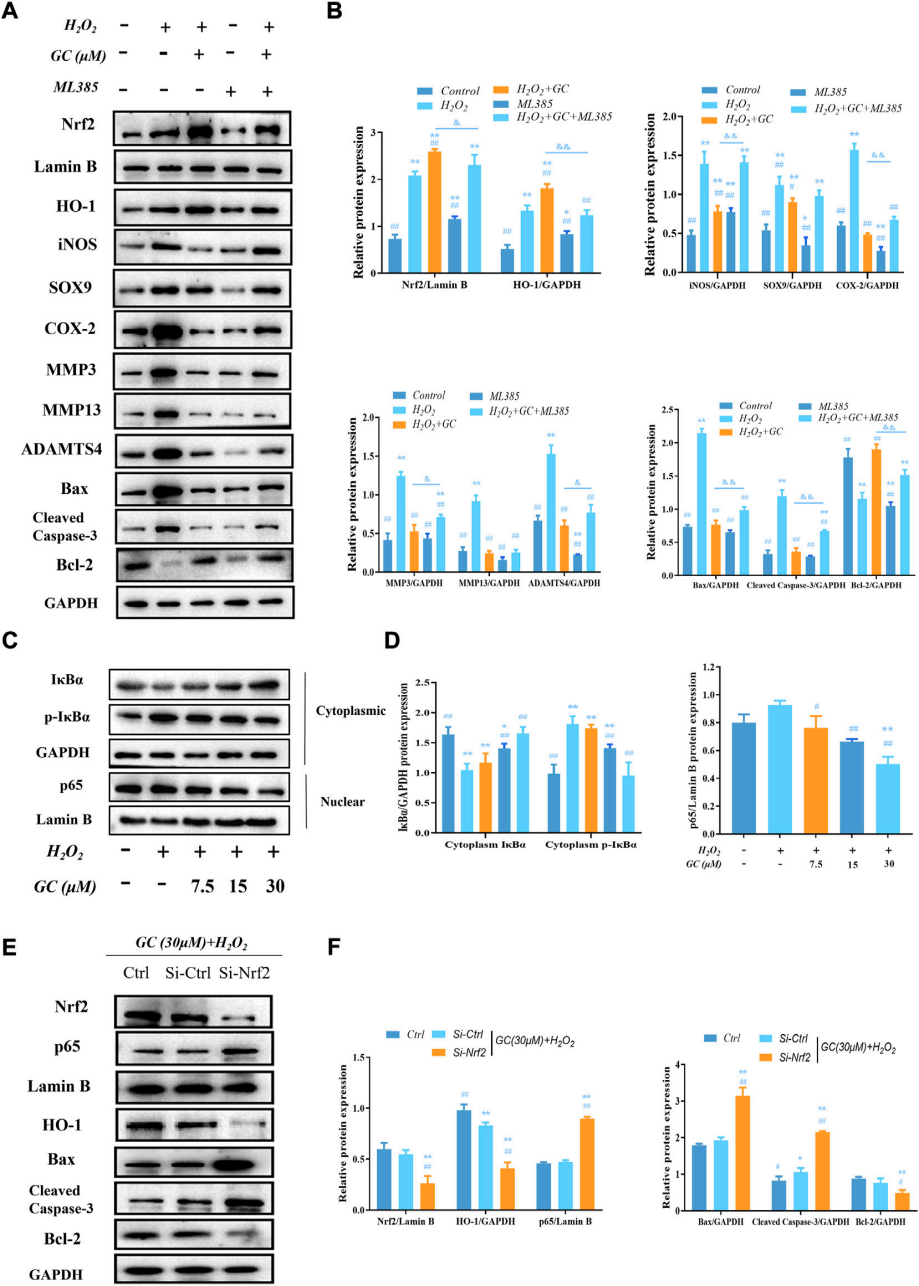
GC exerts protective effect on H_2_O_2_-induced chondrocytes through Nrf2/HO-1 and NF-κB pathways. **(A and B)** Western blot images and quantification data of Nrf2, HO-1, iNOS, SOX9, COX-2, MMP3, MMP13, ADAMTS4, Bcl-2, Bax, and Cleved-caspase three for each group treated with 30 μM GC, H_2_O_2_, and 10 μM ML385. **(C and D)** Western blot image and quantification data for cytoplasm IκBα, p-IκBα, and nuclear p65 protein expression for each group treated with GC and H_2_O_2_. **(E and F)** After transfection with Nrf2 siRNA, the protein expression levels of Nrf2 and p65 in the nucleus and HO-1, Bax, Cleved-caspase 3, and Bcl-2 in the cytoplasmic of chondrocytes co-applied with GC and H_2_O_2_ were analyzed by western blot. All data are presented as mean ± SD (n = 3). **p < 0.01 *vs.* control group; ^##^ *p* < 0.01 *vs.* H_2_O_2_ group; ^＆^ *p* < 0.05 and ^＆＆^ *p* < 0.01 *vs.* H_2_O_2_ + GC group.

Our previous study found that the Nrf2/HO-1 pathway was activated after GC intervenes in H_2_O_2_-induced chondrocytes, and the activated Nrf2 was translocated to the nucleus. In addition, we also found that the anti-apoptotic and anti-cartilage degradation effects of GC were related to the expression of HO-1. Therefore, to investigate whether GC upregulates HO-1 *via* Nrf2, the Nrf2 inhibitor ML385 was used to intervene chondrocytes to evaluate the protective effect of GC on H_2_O_2_-induced chondrocytes. Western blot results showed that 10 μM ML385 alone inhibited the expression of nuclear Nrf2 and HO-1 proteins in chondrocytes. On the other hand, compared with the H2O2 + GC group, the expression of cytoplasmic HO-1 and nuclear Nrf2 in chondrocytes of the 10 μM ML385 + 30 μM GC + H_2_O_2_ group was significantly inhibited. Additionally, as predicted, ML385 reversed alterations brought on by GC in proteins associated with apoptosis and cartilage breakdown (Figures 4A,B). These findings suggest that Nrf2 modulates HO-1 expression in response to H_2_O_2_ stimulation and underlies the protective effects of GC on chondrocytes.

### 3.6 In H_2_O_2_-treated chondrocytes, ginkgolide C blocks the NF-κB pathway

An essential target in the cytotoxic action of ROS is NF-κB. According to our study’s western blot results, the expression of p65 in the nucleus increased in response to H_2_O_2_ stimulation, while the expression of cytoplasm IκBα—a protein that functions as a negative regulator of the NF-κB pathway—was significantly reduced, and the protein expression of cytoplasmic p-IκBα was significantly increased. Compared with chondrocytes treated with H_2_O_2_, nuclear p65 and cytoplasmic p-IκBα protein expression decreased in a dose-dependent manner, while cytoplasm IκBα protein expression increased in a dose-dependent manner after GC intervention (Figures 4C,D). Additionally, the expression of Nrf2 was downregulated using RNA interference (RNAi) against it. Western blot analysis revealed that Nrf2 knockout enhanced nuclear p65 levels while increasing chondrocyte apoptosis (Figure 4E, F). These findings imply that GC can protect chondrocytes by activating Nrf2/HO-1, which prevents p65 from entering the nucleus under pathological circumstances.

### 3.7 Ginkgolide C attenuates the severity of joint pain and cartilage degeneration in anterior cruciate ligament transection rats

**FIGURE 5:**
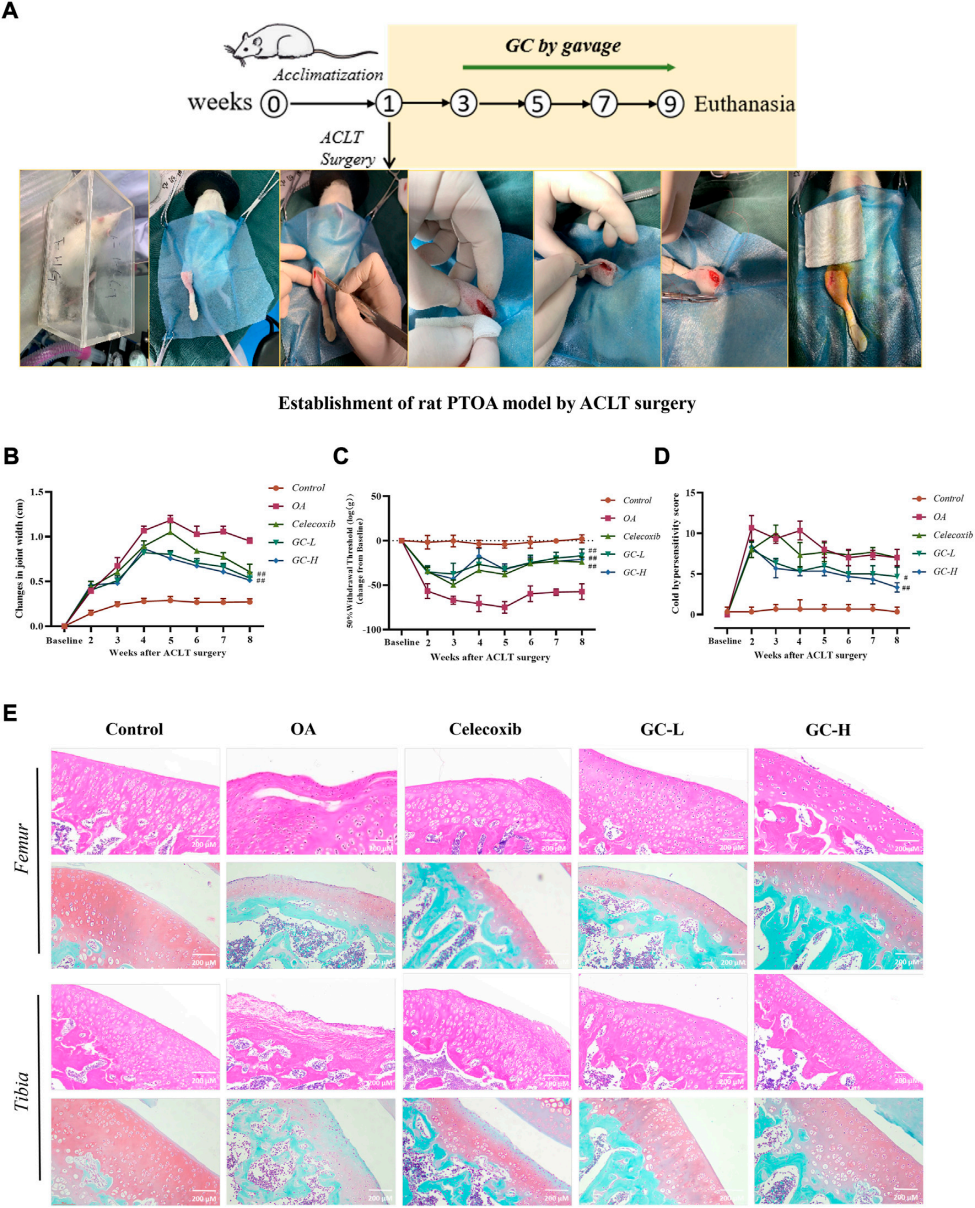
GC attenuates the severity of joint pain and cartilage degeneration in ACLT rats. **(A)** Schematic of the dosing time-course of the rat anterior cruciate ligament transection (ACLT)-induced knee OA model. **(B)** Knee width Measurement. **(C)** Mechanical sensitivity. **(D)** Cold hypersensitivity. **(E)** Representative images of the knee joint stained with hematoxylin-eosin (HE) and Saffron O Fast Green (SO-FG). 200 µm scale bar. All data are presented as mean ± SD (n = 3). *p < 0.05 and **p < 0.01 *vs.* control group; ^#^ *p* < 0.05 and ^##^ *p* < 0.01 *vs.* OA group.

Having established the protective effect of GC on rat chondrocytes *in vitro*, we next examined its ability to relieve pain and reduce cartilage degeneration in a rat model of PTOA. We used one of the most validated models of knee OA, which is the rat ACLT model. ACLT was performed on the right knee of 11- to 12-week-old Sprague-Dawley male rats, and we started GC gavage 2 weeks after ACLT or sham surgery until 8 weeks after surgery, and joint tissue was collected after euthanasia (Figure 5A). The control group did not induce knee swelling, whereas, ACLT surgery-induced OA group showed an increase in knee swelling (Figure 5B). On the other hand, after 8 weeks of gavage GC, the knee swelling significantly reduced when compared to the untreated OA group. Further, we assessed the status of knee pain and discomfort in rats using the von Frey and cold hypersensitivity test (Figures 5C,D). The results showed that changes in right hind limb mechanical sensitivity and cold sensitivity were significantly higher in the untreated OA group than in the GC-treated group, whereas no changes in hind limb pain were observed in the control group.

Next, we examined GC-induced pathological changes in the tibial and femoral cartilage in the ACLT-induced PTOA rat model (Figure 5E). The tibial and femur cartilage surface was smooth, the chondrocytes were orderly distributed, and the SO-FG staining intensity was strong in the control group. In contrast, the chondrocytes in the OA group were severely damaged, and the surface of the articular cartilage was destroyed, indicating severe cartilage damage. Massive ECM loss was seen in the tibia and femur of the OA group, and SO-FG staining intensity dropped. Following celecoxib administration, the cartilage damage was reduced in general, but the femur was delaminated, and the femoral damage remained severe. However, following 6 weeks of experimental treatment of GC, HE staining showed that it improved cartilage surface destruction, stabilized the cartilage microenvironment, and reduced the progression of OA. In comparison to the OA group, the ECM staining intensity of the tibia and femur in the GC-L group and the GC-H group considerably improved. The articular surface became smoother with increasing GC concentration. Notably, both tibia and femur OARSI scores were significantly lower in the GC group than in the OA group (Supplementary Figure S2).

### 3.8 Ginkgolide C improves subchondral bone microstructural changes in osteoarthritis rats, thereby inhibiting abnormal subchondral bone remodeling

**FIGURE 6:**
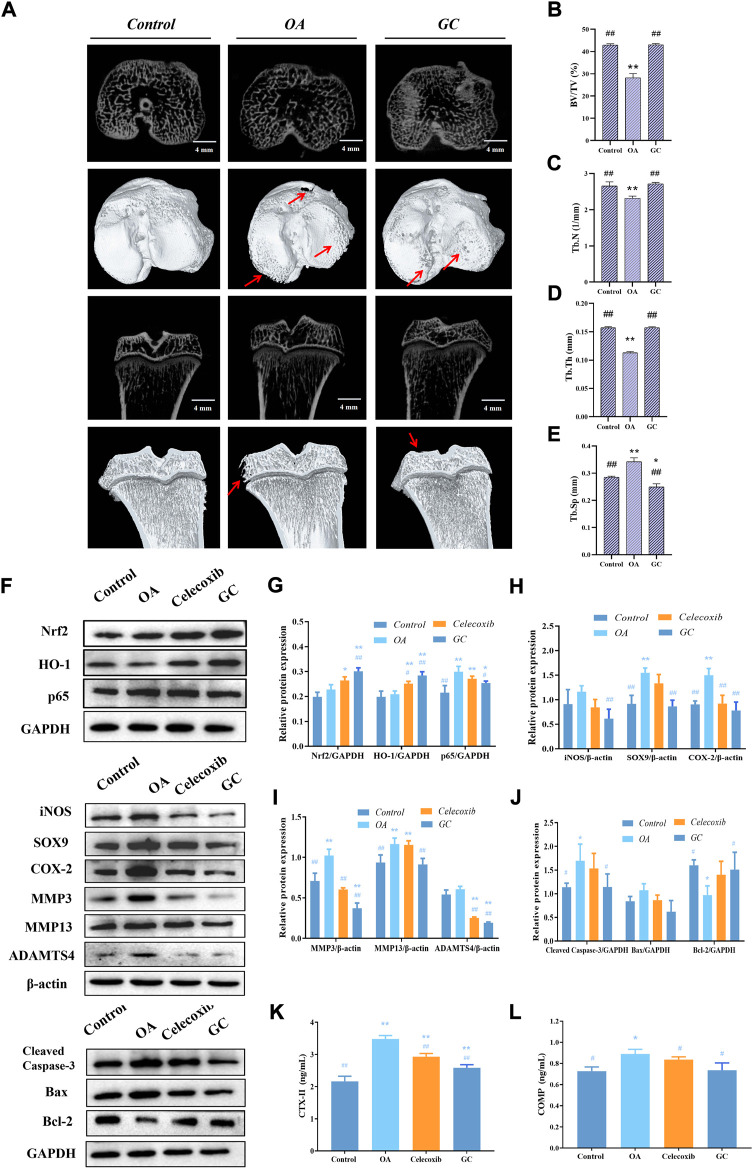
GC regulates Nrf2/HO-1 and NF-κB pathways to rescue joint degeneration in OA rats *in vivo*. **(A)** Microcomputed tomography analysis of proximal tibias from three groups as follows: control, OA, GC (10 mg/kg body weight). The damaged region of the articular surface is indicated by red arrows. 4 mm scale bar. **(B)** Quantitative results of the structural parameters of the tibial subchondral bone, including bone volume/tissue volume (BV/TV), **(C)** trabecular number (Tb.N), **(D)** trabecular thickness (Tb.Th), and **(E)** trabecular separation (Tb.Sp). **(F–J)** Western blot analysis of Nrf2/HO-1 and NF-κB pathway related proteins expression in rat cartilage. **(K–L)** Effects of GC on the levels of biomarkers CTX-II and COMP in serum of PTOA rats. All data are presented as mean ± SD (n = 3). *p < 0.05 and **p < 0.01 *vs.* control group; ^#^ *p* < 0.05 and ^##^ *p* < 0.01 *vs.* OA group.

After demonstrating the inhibitory effect of GC on cartilage degeneration *in vivo*, we wanted to know whether GC has a protective effect on subchondral bone, so we focused on the biomechanical properties and microstructural changes of 10 mg/kg GC on subchondral bone in part (Figure 6A). We observed extensive bone loss in the OA groups based on microcomputed tomography. However, GC was shown to attenuate this ACLT-induced bone loss. The 3D reconstruction results showed that the surface of the tibia in the OA group was uneven, and the bone at the edge of the tibial plateau was eroded, indicating that the structural integrity of the subchondral bone was damaged. The subchondral bone surface roughness of the GC group was improved compared with the OA group. The quantitative results of subchondral bone structure parameters showed that compared with the control group, the bone volume/tissue volume (BV/TV), trabecular number (Tb.N) and trabecular thickness (Tb.Th) parameters of the OA group were significantly decreased, While trabecular separation (Tb.Sp) was significantly increased, the GC group was able to reverse these parameter changes (Figures 6B–E).

### 3.9 Ginkgolide C alleviates osteoarthritis development in a rat model of anterior cruciate ligament transection through Nrf2/HO-1 and NF-κB pathways

As shown in Figures 6F–J, we observed that the expressions of Nrf2 and HO-1 were significantly increased while p65 was down-regulated in cartilage after GC administration compared with the OA group. The expressions of iNOS, SOX-9, COX-2, and pro-ECM degradation proteins MMP3, MMP13, and ADAMTS4 were significantly increased in the OA group, and 10 mg/kg GC could reverse these proteins expression. Notably, 10 mg/kg GC decreased the expression of Cleaved Caspase-3 protein, while the expression of Bcl-2 protein was significantly increased.

The type II collagen degradation product CTX-II in the cartilage ECM, as well as the non-collagen COMP, are key biomarkers for assessing cartilage metabolism (Mobasheri et al., 2017; Posey et al., 2018). According to the results, serum CTX-II and COMP levels were significantly increased after ACLT surgery compared to the control group (Figure 6K, L). Compared with the OA group, serum CTX-II and COMP levels were significantly decreased after GC administration. In addition, celecoxib significantly decreased the levels of cartilage degradation markers CTX-II and COMP. The above *in vivo* results were consistent with the results of chondrocytes, indicating that GC can exert a chondroprotective effect on PTOA rats through the Nrf2/HO-1 and NF-κB pathways.

## 4 Discussion

Inflammatory response and oxidative stress play key roles in the degenerative course of OA (Tudorachi et al., 2021; Katsoula et al., 2022). It has been shown in several investigations that oxidative stress can cause articular cartilage dysfunction (Ziskoven et al., 2010). Therefore, preventing the inflammatory response and ECM degradation brought on by oxidative stress may be crucial in the treatment of OA. Multiple pharmacological actions of GC have been documented, although it is uncertain if these effects might confer protection against OA (Zhang et al., 2018; Yang et al., 2020; Yang et al., 2021). In the present work, we show that GC can suppress the NF-κB pathway and activate the Nrf2/HO-1 pathway, therefore reducing the apoptosis and ECM deterioration brought on by H_2_O_2_. Additionally, *in vivo* research has demonstrated that GC can slow the course of OA in rats.

Numerous variables, such as metabolic stress, inflammatory cytokines, and mechanical loading, which can result in increased ROS levels, are involved in the pathogenesis of degenerative bone and joint disorders (Lepetsos and Papavassiliou, 2016). Abnormally high ROS promote the buildup of damaged DNA and mitochondria, which disrupts cellular equilibrium and is particularly prominent in aging-related illnesses (Collins et al., 2016; Drevet et al., 2018; Hajam et al., 2022). Apoptosis and aging may be caused primarily by excessive ROS production and an imbalance in antioxidant capacity, and the development of OA involves a variety of complicated pathogenic pathways, including apoptosis and ECM deterioration (Yudoh et al., 2005; Hwang and Kim, 2015). As established in a prior research, we employed 0.4 mM H_2_O_2_ as a ROS donor to trigger oxidative damage in chondrocytes *in vitro* (Zheng et al., 2020; Ma et al., 2022b). As expected, H_2_O_2_ reduced chondrocyte viability, promoted inflammatory response and apoptosis, and increased cartilage matrix metabolism, whereas GC corrected these processes, demonstrating that it has a protective impact on chondrocytes exposed by oxidative stress.

Numerous studies have shown that natural products can reduce OA by altering chondrocyte redox balance and inflammatory responses *via* the Nrf2/HO-1 and NF-κB signaling pathways (Zhang et al., 2020; Jiang and Cai, 2021; Zhu et al., 2022). The conformation of Keap1 changes in response to oxidative stress, activating Nrf2 bound to Keap1 in the cytoplasm and dissociating into the nucleus, which promotes the production of HO-1 (Motohashi and Yamamoto, 2004). Following H_2_O_2_- stimulation of chondrocytes, both HO-1 and Nrf2 expression increased in the nucleus, indicating that the Nrf2/HO-1 pathway is activated under oxidative stress but is inadequate to prevent cartilage damage and apoptosis from occurring. This study discovered that GC upregulated the Nrf2/HO-1 pathway, potentially protecting chondrocytes. The HO-1 inhibitor ZnPP, however, decreased the protective effect of GC on chondrocytes. However, treatment with the Nrf2 inhibitor ML385 and Nrf2 siRNA lowered the protective impact of GC and repressed the overexpression of HO-1. These findings imply that activation of the Nrf2/HO-1 pathway mediates the protective effect of GC on chondrocytes exposed to H_2_O_2_.

Cartilage inflammation caused by the NF-κB signaling pathway is the main factor aggravating the pathology of OA (Feng and Wu, 2022). Under normal circumstances, p65 binds to its inhibitory protein IκBα and inactivates the NF-κB pathway. After H_2_O_2_ stimulation, IκBα is degraded and p65 translocates into the nucleus, causing a series of reactions. GC inhibits the expression of p65 protein in the nucleus of damaged chondrocytes and promotes the level of IκBα in the cytoplasm. Furthermore, treatment of chondrocytes with Nrf2 siRNA not only reversed Nrf2/HO-1 pathway activation, but also upregulated the expression of nuclear p65. These findings suggest that GC can block the NF-κB pathway by inhibiting nuclear p65 expression and reducing IκBα breakdown. Similar to the *in vitro* findings, GC improved cartilage dysfunction in OA rats through controlling the Nrf2/HO-1 and NF-κB pathway of cartilage.

**FIGURE 7:**
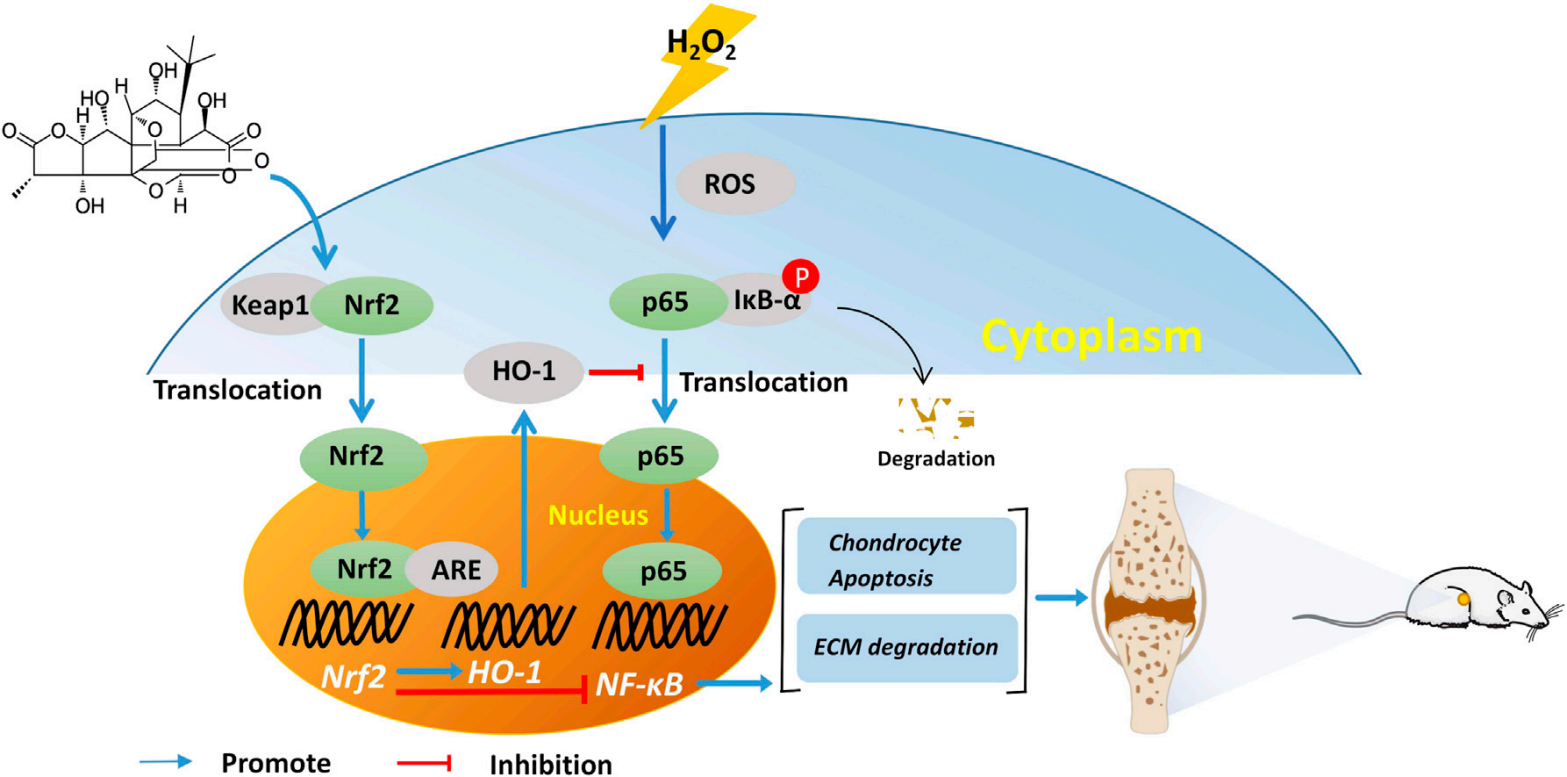
GC slows the progression of osteoarthritis by activating Nrf2/HO-1 and blocking the NF-κB pathway.

The current study showed that GC effectively prevented H_2_O_2_-induced chondrocyte injury by regulating Nrf2 and NF-κB pathways, and exert anti-inflammatory and anti-chondrocyte apoptosis effects to inhibit cartilage degeneration in OA rats (Figure 7). However, these experiments focus on the molecular mechanism by which GC inhibits cartilage degeneration. Therefore, more studies are needed to explain the mechanism of GC on synovial inflammation, subchondral bone remodeling, and pain. Further, the small number of experiments is also one of the limitations of this study, and more large-sample experiments are needed to determine the mechanism of action of GC in the future. Importantly, extensive pharmacological and toxicity studies are required for the treatment of OA with GC before investigating the possibility of its clinical application. However, this study suggests that GC has the potential for treating OA.

## Data Availability

The original contributions presented in the study are included in the article/Supplementary Material, further inquiries can be directed to the corresponding author.

## Abbreviations

OA, Osteoarthritis; ECM, extracellular matrix; GC, Ginkgolide C; H2O2, Hydrogen peroxide; ARE, antioxidant response element; MMP3, matrix metalloproteinase 3; MMP13, matrix metalloproteinase 13; ADAMTS4, thrombospondin motifs 4; iNOS, inducible nitric oxide synthase; COX-2, cyclooxygenase; PTOA, post-traumatic OA; ACLT, anterior cruciate ligament transection; HE, hematoxylin-eosin; SO-FG, Saffron O Fast Green; OARSI, The Osteoarthritis Research Society International; CTX-II, C-telopeptides of type II collagen; COMP, cartilage oligomeric matrix protein; ZnPP, Zinc (II) Protoporphyrin IX; NF-κB, nuclear factor kappa B; Nrf2, nuclear factor erythroid-2 related factor 2; HO-1, heme oxygenase-1; ROS, reactive oxygen species.
