# Supplementary material for: Ginkgolide C slows the progression of osteoarthritis by activating Nrf2/HO-1 and blocking the NF-κB pathway
Source: Front Pharmacol. 2022 Oct 28;13:1027553. doi: 10.3389/fphar.2022.1027553 (PMC9651149; doi:10.3389/fphar.2022.1027553)
Supplement: Supplementary file 1 [file DataSheet1.docx]

Supplementary Material

## Supplementary Figures


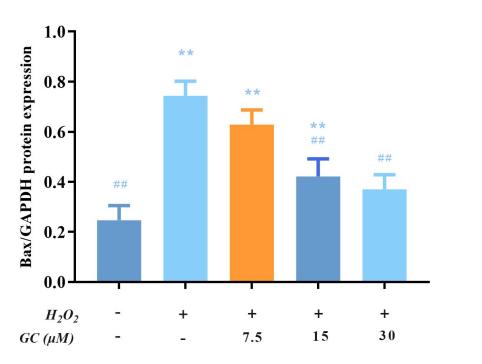

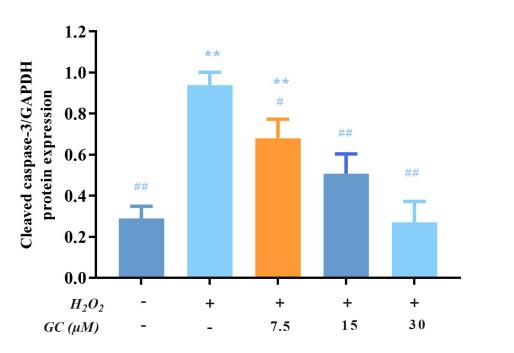

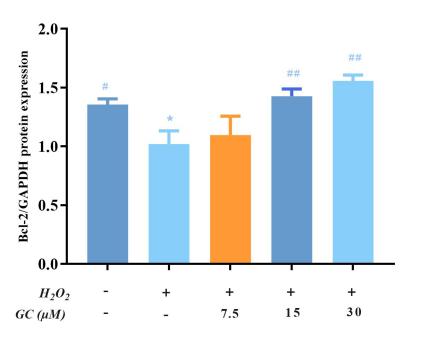


## Supplementary Figure 1. Analysis of Bax, Cleaved-caspase 3 and Bcl-2 protein expression using Image j software. All data are presented as mean ± SD (n=3). * *P* < 0.05 and ** *P* < 0.01 vs control group; ^#^ *P* < 0.05 and ^##^ *P* < 0.01 vs H_2_O_2_ group.

**Supplementary Figure 2.** OARSI score of tibia and femur of rats in each group. All data are presented as mean ± SD (n=3). * *P* < 0.05 and ** *P* < 0.01 vs Control; ^#^ *P* < 0.05 and ^##^ *P* < 0.01 vs OA group.
